# Managing clients’ expectations at the outset of online Cognitive Behavioural Therapy (CBT) for depression

**DOI:** 10.1111/hex.12227

**Published:** 2014-08-02

**Authors:** Stuart Ekberg, Rebecca K. Barnes, David S. Kessler, Alice Malpass, Alison R. G. Shaw

**Affiliations:** ^1^ Institute of Health and Biomedical InnovationQueensland University of TechnologyBrisbaneQldAustralia; ^2^ Centre for Academic Primary CareSchool of Social and Community MedicineUniversity of BristolBristolUK

**Keywords:** Cognitive Behavioural Therapy, conversation analysis, depression, expectation management, first session openings, online therapy

## Abstract

**Background:**

Engaging clients in psychotherapy by managing their expectations is important for therapeutic success. Initial moments in first sessions of therapy are thought to afford an opportunity to establish a shared understanding of how therapy will proceed. However, there is little evidence from analysis of actual sessions of therapy to support this.

**Objective:**

This study utilised recorded session logs to examine how therapists manage clients’ expectations during the first two sessions of online Cognitive Behavioural Therapy (CBT).

**Methods:**

Expectation management was investigated through conversation analysis of sessions from 176 client‐therapist dyads involved in online CBT. The primary focus of analysis was expectation management during the initial moments of first sessions, with a secondary focus on expectations at subsequent points.

**Analysis:**

Clients’ expectations for therapy were most commonly managed during the initial moments of first sessions of therapy. At this point, most therapists either produced a description outlining the tasks of the first and subsequent sessions (*n* = 36) or the first session only (*n* = 108). On other occasions (*n* = 32), no attempt was made to manage clients’ expectations by outlining what would happen in therapy. Observations of the interactional consequences of such an absence suggest clients may struggle to engage with the therapeutic process in the absence of appropriate expectation management by therapists.

**Conclusion:**

Clients may more readily engage from the outset of therapy when provided with an explanation that manages their expectation of what is involved. Therapists can accomplish this by projecting how therapy will proceed, particularly beyond the initial session.

## Background

Service user expectations, and the consequences of these expectations, have been a popular object of study in health services research.^1^, ^2^, ^3^, ^4^, ^5^, ^6^, ^7^, ^8^ One expectation relates to the healthcare process: that is, what a service user will actually do with a professional to address their particular needs.^8^ In countries such as the UK, for example, most people will have some idea of what to expect when they consult a General Practitioner (GP) about an acute medical condition – that their problems will be solicited by their GP, that the provider will initiate a series of questions about history of the problem presented, investigate the problem with methods like a physical examination, deliver a diagnosis and recommend where treatment may be appropriate.^2^, ^9^, ^10^ Such expectations are likely to develop through socialisation across a lifetime of consulting GPs.^11^

In contrast to long‐standing familiarity with GP consultations, people utilising a service like psychotherapy may be unsure about what is involved or could have unrealistic or incorrect expectations.^7^, ^12^ Given that explanations are a fundamental technique to manage the expectations of others,^3^, ^13^, ^14^ this article focuses on how therapists manage their clients’ expectations from the outset of therapy. Examining first sessions of online Cognitive Behavioural Therapy (CBT) for depression, we identify how expectations can be managed by projecting the therapeutic process, as well as exploring problems that can arise when expectations are not managed in this manner. This provides evidence identifying optimal ways of promoting shared understanding of how therapy will proceed.

CBT continues to emerge as the predominant approach to psychotherapy^15^ and is recommended for treating depression in many countries including the UK.^16^ There have been recent attempts to increase access to treatment by developing computerised psychotherapy and online interfaces for therapeutic sessions.^17^, ^18^, ^19^ In both traditional and online therapy, further evidence about the therapeutic process is required to support optimal clinical practice.^20^, ^21^ For instance, initial moments of the beginning of therapy afford an important opportunity to establish shared expectations that may ultimately influence therapeutic outcome.^5^, ^7^, ^12^ It can be particularly important for clients to appreciate that many sessions may be required to achieve therapeutic benefit^22^, ^23^, ^24^ and that initial progress may not therefore be as rapid as they might have anticipated. Although recommendations for opening first sessions are provided in handbooks,^25^ we are not aware of research exploring how this is accomplished in actual sessions of CBT. This article addresses this gap by describing how therapists open initial sessions with clients and the implications for engaging clients in therapy.

The initial moments of first sessions in CBT, which are predominantly focused on assessing a client's situation (hereafter referred to as the ‘assessment phase’), afford opportunities for therapists to explain to clients what therapy will be like. This has been described as orienting clients to the structure of therapy^12^ and is crucial for appropriately involving them from the outset of treatment.^26^ There are two main reasons for focusing on first session openings: first, because the strategies that therapists use to open, structure and manage clients’ expectations are thought to be important for therapeutic success;^7^, ^26^ and second, because how therapy is initiated may influence the relationship between therapist and client.^5^, ^7^, ^12^ Achieving consensus about the tasks and goals of therapy is an important part of therapeutic relationships.^27^, ^28^, ^29^ Clients require a means to appreciate what therapy will involve to maximise the likelihood they will commit to that process. Our aim is to identify ways therapists can utilise, or indeed miss, opportunities to manage clients’ expectations at the outset of the therapeutic process.

## Methods

### Data

This study follows a trial of online CBT for primary care clients diagnosed with depression.^30^, ^31^ This study utilizes typed transcripts of online CBT sessions from 183 client‐therapist dyads. Clients were referred to the trial by their GP if they were between 18 and 75 years old, had been diagnosed with a new episode of depression within the preceding 4 weeks and had not been treated for depression in the previous 3 months. Depression was defined as a score of 14 or more with the Beck Depression Inventory (BDI),^32^ and a diagnosis conforming to the World Health Organization's ICD‐10 classification list.^33^ Patients were excluded if they had history of alcohol or substance misuse, a bipolar disorder or a psychotic disorder, if they were already receiving psychotherapy or if they could not communicate proficiently in English.

During the trial, clients and therapists interacted with one another in real‐time via a secure online website (http://www.psychologyonline.co.uk/). Each client could access up to ten‐hour‐long sessions of CBT from one of 15 therapists; on average, clients attended seven sessions. A random sample of therapy transcripts were independently rated using the Revised Cognitive Therapy Scale (CTS‐R),^34^ which confirmed fidelity to the CBT approach.^30^ As the analysis reported below indicates, however, although these sessions globally conformed to the CBT model, as measured by the CTS‐R, there was variation in the specific techniques used by therapists.

This article focuses on interaction between clients and therapists in first and second sessions of therapy, with particular focus given to ways in which client expectations were managed in the initial moments of first sessions. Transcripts were analysed in the same format as the session logs that were available to clients and therapists. Fragments reproduced here have been modified in two ways. First, names have been replaced with pseudonyms, to protect participant anonymity. Second, line numbering has been added as a reference point. Any typographical errors in the original logs have been retained. The study was approved by a UK National Health Service (NHS) Research Ethics Committee.

### Analytic approach

To study therapy sessions, we used Conversation Analytic (CA) methods to systematically examine interaction between clients and therapists.^4^ CA is well suited to studying healthcare communication,^35^, ^36^, ^37^ including psychotherapy,^38^, ^39^, ^40^ and has also been adapted to the study of online interaction.^41^, ^42^, ^43^, ^44^, ^45^, ^46^ Of particular relevance to our current analytic focus prior CA research has identified particular ways of opening consultations that can impact on the way those interactions proceed.^47^ Similar to this, we identify different ways first sessions of online CBT were initiated and the consequences ensuing from this.

Using a standard CA approach,^48^ first and second sessions for all 183 dyads were systematically examined case‐by‐case. Our primary focus was to identify recurrent ways therapists opened initial sessions and attempted to manage clients’ expectations of therapy. A secondary focus was to determine whether expectations were managed at subsequent points during the assessment phase, which occupied the first and second sessions of therapy. Seven dyads were excluded from further analysis because information about how the first session was opened was missing, resulting in 176 sessions available for analysis. We made collections of different types of expectation management, studying them to determine what they accomplished and the sequential trajectories that could follow. This identified patterned differences between types of expectation management. Due to space constraints, we reproduce just a few instances here to illustrate our findings.

## Analysis

Establishing a therapeutic framework is a task typically undertaken by therapists, and our analysis identifies that it is one that therapists routinely initiate at the very outset of therapy, before they launch the first substantive topic for discussion. The few occasions where clients initiated the first topic reveal the uncertainty faced, by at least some of them, about what to expect from therapy. These occasions are specific evidence of broader uncertainty about psychotherapy that has been highlighted in previous research.^7^, ^12^ For example, Alison (P45) initiated the first topic by asking ‘How do we start?’ and Isabel (P152) by asking ‘Do you ask me questions or do I talk?’ Their questions indicate these clients do not know what to expect from CBT and provide insight into the basic expectations therapists need to manage at the outset of therapy. Unlike institutional interactions such as GP consultations, which most people have experience of across their lifetime,^11^ clients in this study generally had no prior experience of psychotherapy. Analysis of the assessment phase of therapy found that few clients, when asked by their therapist, reported prior experience of psychotherapy generally, let alone the CBT approach more specifically. Managing their expectations for therapy therefore has clear relevance.

Our analysis identified three ways in which therapists managed expectations during the initial moments of first sessions: first, therapists managed clients’ expectations about both the first and subsequent sessions of therapy; second, therapists managed expectations about the first session only; and third, no expectation management was attempted. Some therapists tended to use the same approach in first sessions, while others varied in their approach. In what follows, we explore the three ways in which expectations could be managed in the initial moments of first sessions of online CBT.

### Managing expectations about first and subsequent sessions of therapy

In the first type of expectation management we identified, therapists provided a relatively comprehensive explanation that managed their client's expectation about the first session and projected what would be attempted in subsequent sessions. In such instances, therapists not only described what would occur imminently (e.g. that the therapist would ask a series of assessment questions) but also outlined what would happen beyond that (e.g. that core therapeutic work such as goal setting would probably be deferred to the second session). By projecting what is involved in subsequent sessions, therapists provide clients with information that enables them to appreciate that the initial therapy session can be quite different to subsequent therapeutic work. This understanding is particularly important for clients who do not perceive particular therapeutic benefit from early sessions of therapy, as it enables an expectation that the activities of therapy will progressively shift and that benefit may follow later.

Comprehensive expectation management occurred in 36 of the 176 (20.5%) first sessions in our corpus. The following is one instance. It comes from the beginning of a first session involving a therapist Holly and her client Hannah. In her opening, Holly explains the typical structure of a CBT session before continuing to outline her plan for the first and subsequent sessions.

**Fragment 1 [Online CBT: P60‐T5‐S1]**
01 [Holly]: Hello Hannah
02 [Hannah]: Hi Holly.
03 [Holly]: Welcome to online CBT. Any
04: questions you want to ask at this
05: stage?
06 [Hannah]: No questions at present, Just
07: really nervous.
08 [Holly]: Anything you are particularly
09: nervous about?
10 [Hannah]: Talking about my feelings, not
11: good at it.
12 [Holly]: In CBT we concentrate as much
13: on what you are thinking and
14: doing as how you feel as they
15: are all seen to be interlinked. At
16: the beginning of each session
17: we usually agree an agenda and
18: at the end homework. Today I
19: thought it would be useful to
20: discuss what the main difficulties
21: are and get to know you. A first
22: assessment really. This usually
23: continues in the second session
24: where we agree what you want
25: to achieve in therapy and set
26: your therapeutic goals which we
27: evaluate regularly as we go
28: along. How does that sound?
29 [Hannah]: sounds good.
30 [Holly]: Ok do you want to dive in there
31: then and talk about what brings
32: you here.

Holly's attempts to manage Hannah's expectations for the session have a prospective quality. Her turn beginning from line 12 is constructed as preliminary to further activity.^49,50^ For example, although she mentions discussing Hannah's difficulties (at lines 18–21) she does not, at that point, explicitly ask Hannah to tell her about them. Rather, she projects that an assessment of Hannah's situation will be their initial focus, before explaining that other therapeutic work (e.g. goal setting) will be deferred to the next session. She seeks Hannah's assent to this using a response solicitation (*How does that sound?*, line 28).^51^ Constructing her turn in this way initiates a pre‐sequence, a practice commonly used to support the viability of the action it projects.^50,52^ It is only after Hannah responds affirmatively to the solicitation (line 29) that Holly is in a position to begin the activity, she has projected by eliciting Hannah's difficulties (lines 30–32). Although not all preliminary explanations are constructed in this way, the majority of instances in our corpus are pre‐sequences that occasion a response from clients, thereby explicitly seeking to co‐opt them into the plans for therapy.

Holly's pre‐sequence is an example of a practice commonly employed by therapists in our online CBT data. Not only does it project an imminent course of action for the current session (an assessment phase), it also projects future activities that will extend beyond the current session. It is this feature that is common to this type of opening. As the next fragment shows, although the detail of what is projected may differ, what is common amongst these projections is that they involve managing expectations for future sessions of therapy, in particular that they will involve different activities than those undertaken during an assessment phase. It also comes from the beginning of a first session and involves Pete, a client, and Jenny, his therapist.

**Fragment 2 [Online CBT: P141‐T11‐S1]**
01 [Pete]: Hello
02 [Jenny]: Hi Pete. Welcome to our first
03: appointment! Today's session
04: will allow us to talk about what
05: your current situation is, and the
06: type of support you feel you
07: would like right now. At the end
08: of the session we can make a
09: plan as to how you would like
10: to progress. How does that
11: sound?
12 [Pete]: Wonderful
13 [Jenny]: Great. OK, so could you tell me
14: just a little bit about yourself, just
15: so that I can understand your
16: current circumstances, and also
17: an outline of what you feel you
18: would like some help with right
19: now?

There are notable differences between the projections made in Fragments 1 and 2. For example, in Fragment 1, Holly projects homework as an activity that will be set at the end of the session, whereas in Fragment 2, at the same juncture, Jenny makes no mention of such an activity. What is common between the two projections, however, is that they extend beyond projecting an imminent next action to include a subsequent activity or activities. In Fragment 1, an assessment is projected as a next action and goal setting is projected as a subsequent activity. In Fragment 2, discussion of Pete's current situation (arguably another way of describing an assessment) is projected as a next action and a plan for therapy is projected as a subsequent activity (lines 3–10). By explaining that their initial work together is preliminary to subsequent therapeutic tasks, therapists provide clients with information that may enable them to appreciate that initial therapeutic work differs from subsequent work, an understanding that would be particularly important for clients who do not experience immediate therapeutic benefit. The type of expectation management considered so far has been relatively comprehensive, projecting beyond the task that is to immediately follow to outline a broader trajectory that therapy will follow. However, as we shall show in the following section, most expectation management was not as comprehensive.

### Managing expectations about first sessions only

In the second type of opening we identified, therapists gave some preliminary explanation that managed clients’ expectations about their first session, but did not project beyond that session. This second type was the most common in our dataset, occurring in 108 of 176 (61.4%) first sessions. In these instances, therapists tended to outline what would happen during the first session only without explaining what would happen in subsequent sessions.

An example of this type of expectation management occurs in the following instance, involving a therapist Nicole and her client Janet. In her preliminary explanation, Nicole projects a particular structure for therapy, although unlike Fragment 1 this explanation does not project beyond the current session.

**Fragment 3 [Online CBT: P36‐T3‐S1]**
01 [Nicole]: Hello Janet, how are you this
02: morning?
03 [ ]: Janet Brady has entered the room
04 [Janet]: Hello Nicole I am fine thanks but
05: very slow with keyboard skills!
06 [Nicole]: Don't worry about that. I always
07: tell people not to worry about
08: spelling or grammerotherwise we
09: could spend the whole session
10: checking what we have written is
11: that ok with you?
12 [Janet]: great!
13 [Nicole]: Ok. In this first session I need to
14: get some background
15: information from you that will
16: help me assess you and your
17: problems is that ok?
18 [Janet]: yes happy to supply you with
19: apotted history of my life and
20: living with depression
21 [Nicole]: ok. I will do this by asking you a
22: series of questions. If at anytime
23: you think i am going to quick,
24: you don't understand or you
25: nedd a break, or you don't agree
26: with anything I say please do not
27: hesitiate to tell me. as this
28: therapy is for you. We will work
29: together to find suitable solutions
30: to your problems is that ok?
31 [Janet]: That's fine
32 [Nicole]: Can you confirm your name,
33: date of birth, occupation, marital
34: status, in or out of a relationship,
35: do you have any children and
36: your GP

As in Fragments 1 and 2, Nicole initiates a pre‐sequence to establish, in advance, space to assess Janet's reasons for seeking therapy. However, unlike the earlier instance, Nicole does not project what will happen beyond that assessment. She does not utilise this opportunity to project a range of therapeutic tasks that will take place in future sessions, therefore eschewing an opportunity to outline more broadly the therapeutic process. Nicole does claim that therapy will be collaborative (lines 28–30), but does not provide Janet with details that would enable her to appreciate that subsequent sessions will involve different therapeutic activities from those that are to be undertaken in the first.

Although Nicole does not project beyond the first session, she does nevertheless seek to manage Janet's expectations about what will immediately follow. Nicole initially explains that she will conduct an assessment (lines 13–17) and subsequently explains that she will do so by asking a series of questions (lines 21–22). She also uses this opportunity to explain to Janet that this activity can be interrupted for a range of reasons (lines 22–28). On two occasions, at lines 11 and 30, she seeks Janet's assent to her projected plans. In this way, Nicole seeks to manage Janet's expectations for their imminent work together. A similar practice of managing expectations about the imminent future is used in Fragment 4, involving Paul, a client, and Stephanie, his therapist.

**Fragment 4 [Online CBT: P51‐T4‐S1]**
01 [Paul]: Good Morning
02 [ ]: Stephanie Moore has entered
03: the room
04 [Stephanie]: Hello Paul
05 [Paul]: Hi
06 [Stephanie]: Welcome
07 [Stephanie]: Perhaps we could start off the
08: session today with you telling me
09: a little bit about yourself and
10: what has brought you to have
11: some CBT (cognitive behaviour
12: therapy). How does that sound?
13 [Paul]: Sounds good to me.
14 [Paul]: Erm How to begin is a tough
15: one, ((continues))

As in Fragment 3, here Stephanie projects (at lines 7–12) a particular course of action that she and Paul subsequently engage in. Unlike in Fragments 1 and 2, her projection does not extend beyond the imminent next action to outline activities the dyad will engage in subsequently. The activity is constructed, however, as time‐limited. Stephanie suggests that Paul's description of himself and his reason for seeking therapy will ‘start off the session’ (lines 7–8). In this sense, there is a means for Paul to appreciate that at least a further activity, if not activities, will follow his initial description. Nevertheless, Stephanie's projection provides no details of what subsequent activity will be. This is the crucial difference between the two types of action projections we have considered so far.

Explanations are a method for managing the expectations of others,^3,13,14^ and the two types of explanation considered above illustrate how therapeutic process can be projected to varying degrees. This may have consequences for the subsequent interaction between therapist and client and the longer‐term progress of therapy. A more immediate consequence of expectation management, however, is that it appears to facilitate smooth progress to the therapist's assessment of their client's situation and circumstances. This consequence is apparent in instances where explanations are not produced and expectations are not managed.

### No expectation management at the outset of therapy

One way to appreciate how explanations manage clients’ expectations is by observing occasions where this does not occur. In the final type of therapy opening we identified, therapists ask a therapy‐oriented question without first attempting to manage clients’ expectations about what therapy will involve. This type was identified in 32 of 176 (18.2%) first sessions in our corpus. Only two of the 15 therapists in our study opened first sessions in this manner. Where this did occur, however, it often occasioned a disavowing (that is, a ‘non‐answering’) response from clients. Although uncommon, these instances are useful ‘deviant cases’^53^ to identify the value of expectation management. The following is one such instance. It involves Stephanie, the same therapist as in Fragment 4, and her client Jennifer. As with the above fragments, it comes from the beginning of a first session of therapy.

**Fragment 5 [Online CBT: P53‐T4‐S1]**
01 [Jennifer]: hello stephanie I am early just to
02: make certain everything goes
03: according to plan. the time is
04: 7.40.
05 [Stephanie]: Hello Jennifer
06 [Stephanie]: glad things have gone smoother
07: this time.
08 [Jennifer]: hi i am here
09 [Stephanie]: how can i help?
10 [Jennifer]: oh I don't know hoping you
11: would have all the answers
12 [Stephanie]: what kind of situations are
13: problematic for you at the
14: moment?

Following discussion of some apparent difficulty with an earlier session (lines 1–4), Stephanie moves to initiate the business of therapy. Instead of the explanations observed in the earlier fragments, however, Stephanie directly proceeds to seek information. Her question (*how can i help?* line 9) is formatted as a general enquiry.^47^ Although such questions are readily answerable in GP consultations,^47^ a type of institutional encounter most people have experience of,^11^ this question can be difficult for psychotherapy clients to answer, which is further evidence that they may have unclear expectations about therapy. This is indeed the case for Jennifer, who replies with a disavowing response (lines 10–11). She treats Stephanie's question as anticipating that she will be able to articulate how psychotherapy can help her. By typing ‘hoping you would have all the answers’, Jennifer defers responsibility for this to Stephanie as her therapist. Jennifer's disavowing response puts Stephanie in the position of having to attempt to begin the business of therapy all over again, which she does with a more specific question at lines 12–14.

The opening moments of the session in Fragment 5 lack key elements observed in previous fragments. By projecting what will happen in the first session, and perhaps beyond, therapists provide clients with means to understand how they should contribute to the therapeutic process. In contrast, with no expectation management, clients have little structure to appreciate how they can contribute. It is important to be clear, however, that this is not necessarily the case. Although Jennifer struggled to respond to Stephanie's question, the following fragment shows a client, Danielle, who displayed no difficulty responding to a near identical question from her therapist Tim. Prior to the beginning of the fragment, Tim has been explaining confidentiality and aspects of the online modality that they are using to interact with one another, but has not yet moved into the assessment phase of the session.

**Fragment 6 [Online CBT: P17‐T2‐S1]**
25 [Tim]: Okay. So, how can I help you?
26 [Danielle]: Well – my life is one big mess. I
27: am now a house wife looking
28: after 3 children. One at school
29: and two liyyle ones at home. I
30: should be on top of things but I'm
31: not. I can't seem to cope with
32: everyday things like cleaning,
33: ironing etc., The day seems to
34: go by and I haven't got these
35: things done. As the months have
36: by this is starting to upset me
37: more and more… I also have
38: the most terrible mood swings. I
39: would like to sort myself out and
40: go back to the kind, patient
41: person that I once was.
42 [Tim]: Tell me about the person you
43: once were?

Danielle's response to Tim's question displays that she has some understanding of her role in the therapeutic process. Her understanding is that her role is to articulate the current problems in her life and the change she seeks to achieve. Irrespective of whether Danielle's understanding is appropriate, a comparison of responses in Fragments 5 and 6 suggests that clients bring different levels of expectations to psychotherapy. Although general enquiries that are not prefaced with preliminary explanations will not always occasion disavowing responses, this is nonetheless a risk faced by therapists using this approach. In the absence of some form of preliminary explanation, clients may not appreciate that therapists’ initial questions are part of a process, they may not understand their role in that process, and they may not identify how it might benefit them.

In summary, most therapists did attempt to manage clients’ expectations at the outset of therapy. Such attempts typically oriented the client to the process of the first session, sometimes projecting beyond to future sessions, thereby managing clients’ expectations of the therapeutic process more broadly. Our analysis suggests opening a first session of therapy with some expectation management is more beneficial for the therapeutic interaction than opening a session without such an explanation. Initial moments of first sessions provide a unique opportunity to manage clients’ expectations. As we shall show in the following section, therapists are far less likely to manage expectations during the remainder of the assessment phase.

### Subsequent expectation management

In addition to examining the initial moments of first sessions, our analysis also included an inspection of the entire assessment phase of therapy. The aim of this examination was to evaluate the extent to which expectations about therapy are managed before therapists and clients move from an assessment of the client's situation to specifically addressing aspects of the client’s situation that may be contributing to their distress. Given the assessment phase sometimes extended into the second session, our analysis of a dyad continued until a clear move had been made from the assessment phase to the standard session format that defined the subsequent sessions of therapy.^25^ Our analysis identified that, in principle, therapists could manage client's expectations at a variety of points during the assessment phase. The first session between a therapist Nicole and her client Fiona is an example of this. In addition to managing the Fiona's expectations during the initial moments of therapy, Nicole also provides additional explanation of the therapeutic process during the closing moments of that same session, immediately after Nicole and Fiona have arranged their next meeting. The following fragment shows this expectation management at both the beginning and end of the session.

**Fragment 7 [Online CBT: P43‐T3‐S1]**
001 [Nicole]: Hello Fiona, how are you this
002: morning?
003 [ ]: Fiona Russell has entered the room
004 [Fiona]: Hi Nicole, I am fine thank you
005 [Nicole]: Great! In this first session I need
006: to get some background
007: information from you that will
008: help me assess you and your
009: problems. I will do this by asking
010: you a series of questions, is that
011: ok?
012 [Fiona]: yes, that will be ok
 ((181 lines omitted))
192 [Nicole]: ok I want to say to you thank
193: you for working very hard and
194: next week we will finish the
195: assessment and start the work
196: on the therapry. Take care and
197: speak next week bye for now
198 [Fiona]: Bye

Nicole is the same therapist as considered in Fragment 3. Here, in her session with Fiona, she uses a similar explanation at lines 5–10 as she provided to Janet at lines 13–17 of Fragment 3. Both explanations provide a means for managing clients’ expectations about the activity that is to follow, but they do not outline subsequent activities that will constitute the therapeutic process. In her session with Fiona, however, Nicole provides additional information about the therapeutic approach to that outlined during the initial moments of therapy. At the end of the session, she explains that in their following session, they will complete their assessment of Fiona's situation before moving to commence therapy (lines 192–197). Just as expectation management during the initial moments of first sessions may help orient clients to the structure of therapy, expectation management at subsequent points provides further opportunities for clients to understand the process and trajectory of therapy.

Although not common, there were other occasions like the above instance involving Fiona and Nicole. In 27 of 176 dyads (15.3%), expectations were managed at some point after the opening moments of the session. In 20 dyads (11.4%), the therapist had already managed the client's expectations in the initial moments of the first session. Fragment 7 is an example of this. In only seven dyads (4.0%), however, were expectations managed at a subsequent point in the assessment phase but not during the initial moments of the first session. Our analysis therefore reveals that most common point during the assessment phase at which clients’ expectations are managed is during the initial moments of the first session. At this point in the interaction, 144 clients (81.8%) had their expectations managed to some extent. The initial moments of therapy therefore afford a critical opportunity for therapists to explain the process of therapy to their clients.

## Discussion

Aligning the expectations of clients and healthcare providers regarding their work together is an important factor in treatment success and client satisfaction.^5^, ^7^, ^8^ This article addresses one component of this, examining how healthcare professionals can manage clients’ expectations about the treatment process. Focusing on online CBT for depression, we explored ways in which therapists manage clients’ expectations at the outset of therapy. On this basis, we distinguished initial moments of first sessions into three types. In the first type, therapists gave relatively comprehensive projections of the activities involved in therapy. This involved describing activities that would constitute the first session, as well as projecting what would be involved in subsequent sessions. In the second type, therapists outlined what would happen in the first session, but did not mention what would happen in subsequent sessions. In the third type, therapists made no attempt, in the initial moments of the first session, to manage clients’ expectations about the therapeutic process.

Our analysis also identified evidence in support of managing clients’ expectations at the outset of therapy. First, occasions where therapists made no attempt to manage clients’ expectations were liable to occasion difficulties. Most commonly, difficulties involved clients displaying uncertainty about how to respond to their therapist's first assessment question. Initiating the process of expectation management at the beginning of therapy is a clear way for therapists to enhance the likelihood that clients will engage in the therapeutic process from its outset. It is also an opportunity to convey that clients may need to remain in therapy for many sessions to derive an optimal therapeutic benefit.^22^, ^23^, ^24^ Finally, given that people can hold themselves, and one another, accountable to explanations they provide,^13^ managing expectations at the outset of therapy may help to make both therapists and clients accountable to the process they have agreed to follow.

This study follows calls for evidence‐based explanations of the psychotherapeutic process that can be used to improve treatment.^20^, ^21^ Although there are suggestions for how therapy sessions should be opened and clients’ expectations managed,^7^, ^12^, ^25^ we believe this is the first attempt to observe how this is accomplished in actual sessions of psychotherapy. We explore the local consequences of using different ways of opening first sessions of online CBT, finding those that project a process are more likely to result in productive responses from clients. Some therapists consistently used the same approach in first sessions, while others varied in their approach. In another article, we report a quantitative study based on the analysis provided here that shows managing expectations from the outset of therapy is associated with increased retention of clients in online CBT.^54^

More broadly, our study highlights ways in which different types of healthcare encounters can require managing clients’ expectations in different ways. For example, although existing research has identified that service users visiting a GP can readily answer general enquiries,^47^ our research demonstrates that people may struggle to answer to the same type of question when asked in a different institutional setting like online psychotherapy. It is likely that such questions are more readily answered in settings such as primary health consultations, as service users have been socialised into the process across a lifetime of encounters.^11^ Such extensive socialisation is unlikely to be the case, however, for the vast majority of clients attending psychotherapy. They may have no prior experience of therapy, or their experience may be with a different therapeutic approach. This highlights important institutional differences can exist that impact on expectation management. Our research suggests managing expectations is particularly important for types of healthcare services that clients do not routinely visit. It is also important to consider differences in levels of expectations that are likely to exist between clients and to manage these accordingly.

Managing clients’ expectations is important across different types of healthcare encounters, although it appears the manner in which this is attempted differs across different types of encounters. In online CBT, we find that managing expectations at the very outset of therapy is a means to circumvent initial problems in engaging clients in the therapeutic process. More broadly, all healthcare providers should consider appropriate ways of managing their clients’ expectations about the consultation and treatment process.

## Conflict of interests

No conflict of interests have been declared.

## Source of funding

This research was supported by a grant from the Bupa Foundation (London, UK).
